# The Eye as a Window to Brain Health: Can Retinal Imaging and AI Modeling Predict Alzheimer's Disease?

**DOI:** 10.1002/brb3.70890

**Published:** 2025-10-01

**Authors:** Eniola Awodiya

**Affiliations:** ^1^ Green Templeton College University of Oxford Oxford UK

**Keywords:** AI, Alzheimer's, retinal imaging

## Abstract

**Objective:**

This is a review article to evaluate clinical evidence for the vascular model of Alzheimer's disease (AD) pathology and elucidate the extent to which retinal imaging with AI modeling can be useful in earlier diagnosis of the condition.

**Methods:**

I comprehensively reviewed the literature on the current understanding of AD pathology and emerging role of the neurovascular system. I reviewed the evidence for retinal vascular biomarkers that can predict the presence of cognitive impairments seen in AD and AI models that utilize these to diagnose and elucidate pathophysiology for the condition.

**Results:**

It is found that retinal imaging offers a non‐invasive and cost‐effective way to detect AD‐related neurovascular changes and, when coupled with AI, holds a transformative role in improving screening, diagnostics, and our understanding of the disease.

**Conclusion:**

There is evidence to suggest that retinal imaging can provide an earlier diagnosis of the condition. This can change the practice by encouraging lifestyle modification as crucial in modifying the progression of the disease.

## Graphical abstract

Our growing understanding of Alzheimer's disease pathophysiology involves neurovascular dysfunction. There is evidence to suggest this can be visualized non‐invasively through retinal imaging. This review explores how artificial intelligence enhances the detection of retinal imaging biomarkers to enable earlier diagnosis, improved understanding of disease mechanisms, and timely lifestyle interventions for this disease.

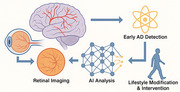

## 1 Introduction

Alzheimer's disease (AD) is the most common of the dementias (Oh and Rabins 2019), characterized by disorder in memory, cognition, executive processing, personality, and behavior (Lyketsos et al. 2011). With the increasing aging population worldwide, its prevalence has grown significantly in the last few decades. Studies reported the disease burden to be at 44 million in 2016, an increase of 117% since 1990 (Lyketsos et al. 2011). Of significance, however, the trend in the development of dementia is shown to be higher in low‐ and middle‐income countries, suggesting the relevance of socioeconomic determinants of health in the prevention, and management, of AD. In parts of Africa, dementia, including AD, poses significant challenges due to limited healthcare infrastructure and socioeconomic barriers (Kantawala et al. 2023), highlighting a growing need for non‐invasive screening methods like retinal imaging. There is also some evidence that many of the significant risk factors for the disease, such as smoking (particularly in men) and obesity (particularly in women), are in fact modifiable. This posits the question of whether earlier detection and effective wide scale screening, coupled with policy changes, can address the disease burden currently caused by this condition.

The eyes being the window to “something more” is not a new concept. The famous quote “the eyes are the window to the soul” highlights the philosophy that the eyes, unlike any other organ, can convey more about a person's well‐being and emotional state than what is originally portrayed. Biologically, however, this quote extends to hold some relevance. The eye can be seen as an anatomical extension of the brain, with the embryological process of neurulation where development of the brain and spinal cord occurs, underpinning the development of overlapping structures between the systems (Bales et al. 2025). Many of the disease processes affecting the brain therefore map onto the eye in a similar way, particularly neurodegenerative processes (Nguyen et al. 2021). As a key place in the body where both neurons and blood vessels can be imaged concurrently, ophthalmic tests have already become central to the monitoring of systemic disease, such as diabetes, and pose an exciting, non‐invasive, investigative tool for other neurological conditions.

In this essay, I will first seek to explore the current models for the pathophysiology of Alzheimer's dementia and posit how ophthalmic investigations may be of diagnostic utility in this often mischaracterized condition. I will then address the inherent connection between the eyes and the brain, and explore how retinal imaging can be a novel, cost‐effective, and non‐invasive method of screening for Alzheimer's related pathology. I will then end by looking into how the rising efficacy of AI models can be coupled with this, and health policy changes, to revolutionize the screening for, diagnosis of, and prevention of Alzheimer's dementia.

### 1.1 Our Understanding of the Pathophysiology of Alzheimer's Disease Has Changed

Traditionally, AD has been viewed through the amyloid‐beta (Aβ) hypothesis and tau protein accumulation models. However, emerging research suggests a crucial role for vascular dysfunction. I will now explore the evidence for these three models.

### 1.2 Alzheimer's Disease as a Tauopathy

One of the models of the pathophysiology of AD is a disorder of intracellular processes involving tau aggregates forming neurofibrillary tangles. These in turn impede cellular functions, such as axonal transport.

This idea began in the early 1900s with the latest techniques in histochemistry allowing for the detection of multiple plaques and tangles in the postmortem brains of 16 patients with senile dementia (Stelzmann et al. 1995), and later by Alzheimer in 6 patients. Eighty years later, major components of these tangles were found to be comprised of hyperphosphorylated, filamentous tau (Grundke‐Iqbal et al. 1986). This was furthered by the discovery that the microtubule‐associated protein tau (MAPT) gene causes a fronto‐temporal dementia with Parkinsonism, which added genetic evidence that dysfunction in tau was sufficient in the absence of Aβ to cause neurodegeneration (Spillantini et al. 1998).

It is currently thought that the normal function of tau involves the formation and maintenance of the cellular microtubule structure (Khatoon et al. 1995). However, when hyperphosphorylated, these tangles instead inhibit microtubule assembly, leading to the degradation of neurons, starting first in the trans‐entorhinal cortex, association isocortex, and the primary sensory cortex (Cho et al. 2016), which correlates with cognitive decline.

### 1.3 Alzheimer's Disease as an Amyloidopathy

Another, and slightly more pervasive, model involves the aggregation of amyloid plaques as an extracellular process leading to AD. The first of the categories of evidence supporting this theory includes genetic analyses, showing that the βAPP protein found on chromosome 21 was central to the sporadic form of the disease (Castellani et al. 2024). This is further corroborated by the established increased incidence and earlier onset of AD in patients with Down's syndrome, where trisomy 21 occurs.

Studies further helped to elucidate the mechanism of this pathology. In brain slice preparations of PDAPP mice (transgenic mice models overexpressing mutant human amyloid precursor protein V717F), APP was shown to cause a loss of long‐term potentiation, damage synapses, and kill neurons with selective neurotoxicity to the entorhinal cortex and hippocampus (Larson et al. 1999). Furthermore, injecting Aβ oligomers into mice hippocampi was shown to induce Alzheimer‐like effects such as inflammation, oxidative stress, and tau phosphorylation (Zussy et al. 2013).

APP codes for a transmembrane receptor found in high concentrations in neurological tissue (Müller et al. 2017). In the healthy brain, this protein is cleaved by several enzymes. However, a specific form of this enzyme (Β and γ secretase) cleave APP to form the malignant form, β‐amyloid, which contributes to Alzheimer's by forming aggregates that are directly cytotoxic to neighboring cells.

Given the popularity of these models to explain the basis of AD pathology, these molecular targets have been the focus of exciting new therapeutic agents. The evaluation of these can further help to elucidate the veracity of their role in the condition. Lecanemab is a monoclonal antibody designed to preferentially target soluble Aβ protofibrils, with activity at insoluble fibrils, before they build up to form neurotoxic tangles in the extracellular space (Lord et al. 2009).

**FIGURE 1:**
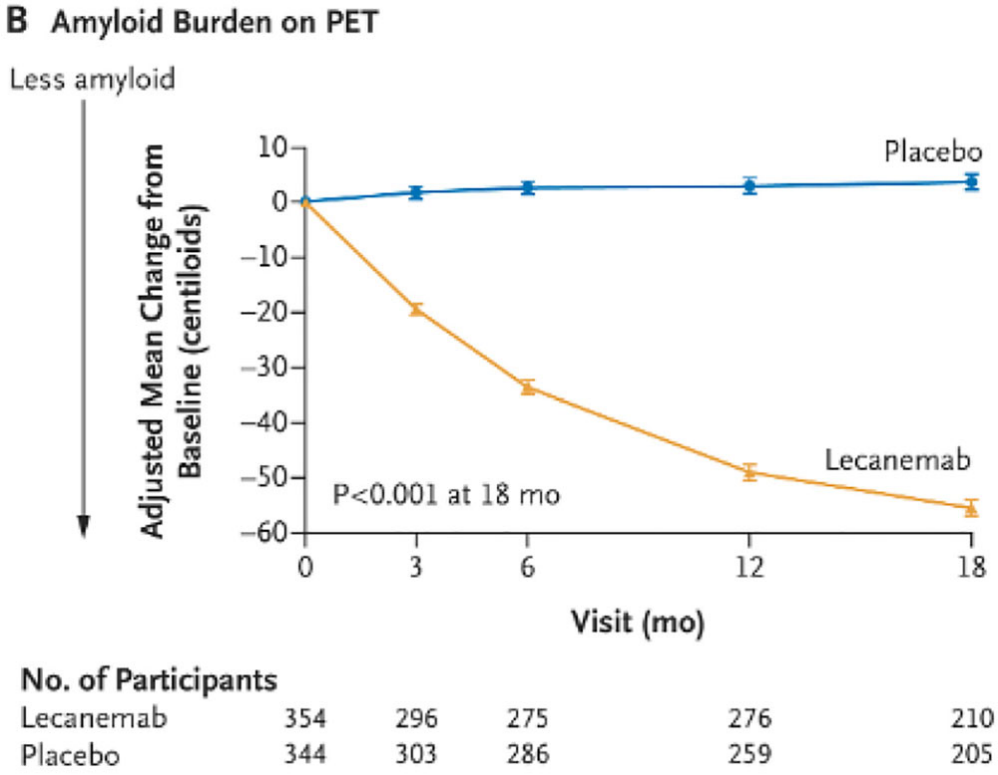
PET‐amyloid burden reduced by 63% with lecanemab.

**FIGURE 2:**
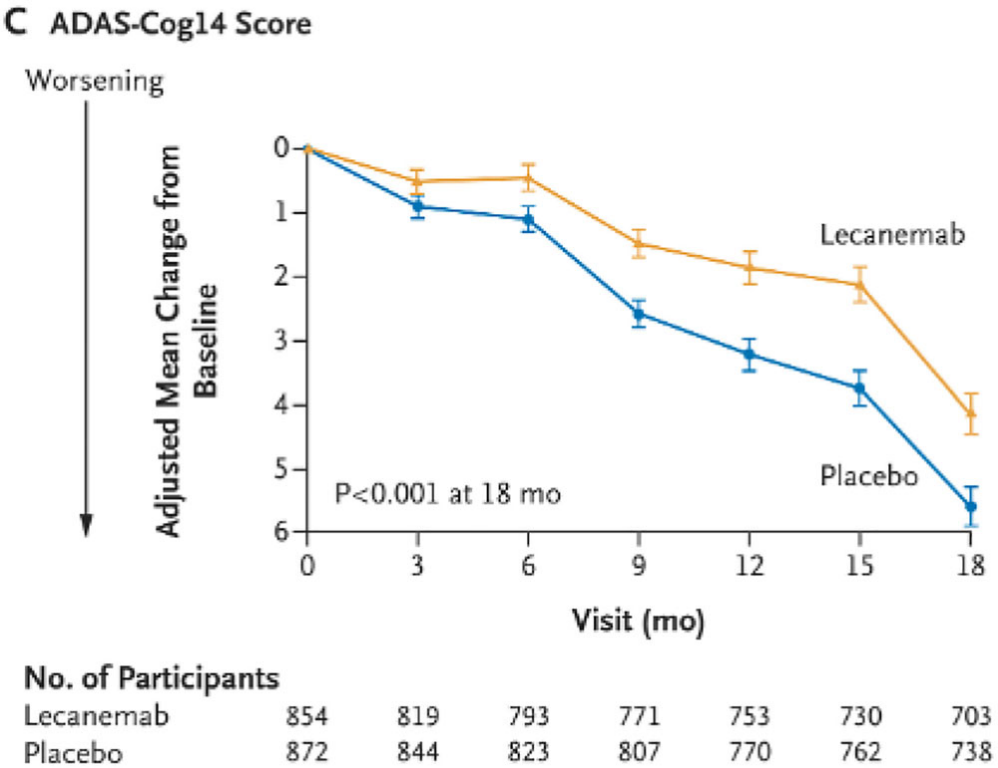
Decreased rate of worsening cognition by 26% on lecanemab.

**FIGURE 3:**
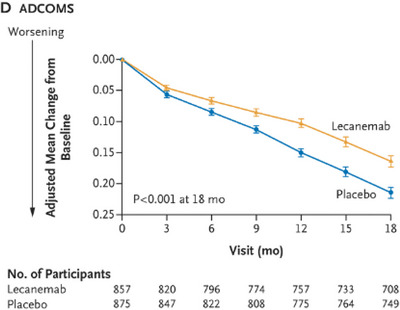
Disease progression slowed by 24% on lecanemab.

**FIGURE 4:**
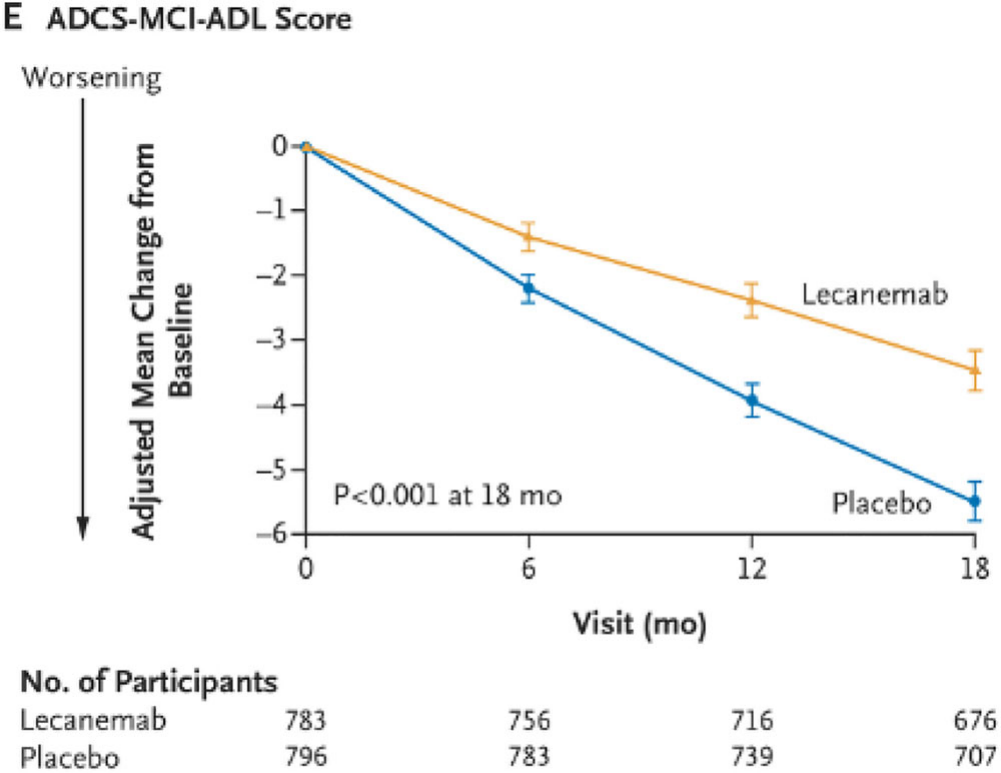
Functional decline slowed by 37% on lecanemab.

The Clarity trial (van Dyck et al. 2023) was an 18‐month multicenter, double‐blinded, placebo‐controlled, and parallel‐group trial involving persons with early AD. Those eligible were stratified to a random group according to ApoE carrier status, geographic region, and the absence or presence of concomitant approved medication for AD. Based on this, participants were given either IV lecenemab (10 mg/kg every 2 weeks) or placebo. Notably, however, while lecanemab was found to significantly lower amyloid, this correlated with only a moderate improvement in cognitive function. Over 18 months, while amyloid burden on PET imaging had dramatically reduced by around 68% (*p < *0.001) (Figure 1), the impact on functional symptom burden was modest in comparison: cognition loss as measured by the ADAS‐cog4 reduced by 26% for those on lecanemab (Figure 2), disease progression was slowed by 24% compared to placebo as measured by the ADCOMS test (Figure 3), and functional decline was slowed by 37% for those on lecanemab as measured by the ADCMS MCI‐ADL test (Figure 4). Worth noting is that the tests used as a measure of disease impact have their limitations which should be considered when interpreting these findings. Some of these are highlighted in Table 1.

**TABLE 1:** Limitations of AD screening tools used.

ADAS‐cog 4 test	ADCOMS test	ADCMS MCI‐ADL
Provides a broad measure of cognitive function, especially in domains impacted early on in AD such as memory, language, and orientation. Subject to ceiling and floor effects, and so lacks the range to detect meaningful performance differences between candidates. Helpful to look at change from baseline score, but results may underrepresent marginal differences in score.	Derived from four items of the ADAS‐cog test, two from the MMSE, and all six from the clinical dementia rating sum of boxes. This makes it a more comprehensive assessment of the disease process, but may miss executive categories we are still unaware of and does not address functional impact.	Focuses on ability to complete activities of daily living as a functional assessment of the impact of the disease. Helpful in conjunction with the other tests for a holistic view of disease progression, but it is subjective and unclear in the data whether collateral or caregiver input was prioritized to ensure validity.

This demonstrates a role for amyloid build‐up in the pathogenesis of AD, but also highlights that the pathology is more multifactorial than this hypothesis alone. Despite the drastic clearance in the burden of this molecule in brain tissue as achieved by this intervention, clinical outcomes fail to reflect this level of change. It appears there is more to uncover in our understanding of the pathology of AD.

### 1.4 Alzheimer's Disease as a Vascular Disease

Again, the identification of a significant genetic component to AD gave rise to another model for its pathology. In 1993, a group at Duke University identified a DNA region linked to a higher risk of late‐onset AD (the most common form of the disorder). The product of this DNA region, the ApoE protein, was found to be present in many of the amyloid plaques involved in the damage caused by AD. Comparison of affected patient genotypes with unaffected relatives further pinpointed the ApoE4 variant as significant (Corder et al. 1993).

There is emerging evidence to support the role of *ApoE4* in the vascular dysfunction model of AD, such as reduced cerebral blood flow, endothelial dysfunction, and the breakdown of the blood–brain barrier (BBB). Knock‐in mice models showed an increase in the BBB's susceptibility to injury by activating the proinflammatory cytokine cyclophilin A in pericytes (Bell et al. 2012). Mice models have also demonstrated 16% and 13% reductions in cerebral blood flow to the cortex and hippocampus, respectively, as well as 17% and 11% reductions in cortical and hippocampal capillary length between knock‐in mice and *ApoE3* littermate controls (Montagne et al. 2021).

These processes are thought to precede the accumulation of toxic aggregates such as β‐amyloid and tau. This has led to a “two‐hit hypothesis” in which primary vascular dysfunction creates an impaired clearance of toxic Aβ and tau, the aggregation of which induces neurotoxicity and cortical atrophy (Eisenmenger et al. 2023).

Further evidence for a vascular model of the disease involves the shared risk factors between the two conditions. In sporadic AD, modifiable risk factors known to increase the risk of disease include high blood pressure, not meeting the aerobic physical activity guideline, obesity, diabetes, depression, current cigarette smoking, hearing loss, and binge drinking (Omura et al. 2022). Analyzing the data from a survey given to those > 45 years old to assess behavioral impact demonstrated that, among those factors, the prevalence of hypertension was found to be highest (49.9%), closely followed by reduced physical activity (49.7%) and obesity (35.3%). Interestingly, one study reports that ApoE has a similar correlate to AD as T2DM, more so than other reported risk factors, with hazard ratios of 1.98 and 1.77, respectively (Gottesman et al. 2017).

This research has culminated in the recent assessment of semaglutide, a GLP‐1 analogue currently used in the management of T2DM, as a potential therapeutic target for AD. Using a US‐wide database of electronic health records, one study looked at over 1 million patients with diagnosed T2DM and no prior AD diagnosis to compare semaglutide with seven other diabetes medications and review whether AD occurred within a 3‐year follow‐up period. Interestingly, semaglutide showed a significant risk reduction for first‐time AD diagnosis when compared to insulin, with a hazard ratio of 0.33, with similar findings demonstrated across obesity status, gender, and age groups (Wang et al. 2024). The study used propensity score matching to account for the possible effect of differences in age, ethnicity, socioeconomic determinants, and pre‐existing conditions, highlighting the significance of the impact of this intervention in outcomes. Preclinical studies have suggested that the proposed mechanism of action for this drug's effects on AD outcomes includes a reduction in Aβ plaques and tau tangles, enhanced autophagy, and increased glucose uptake (Wang et al. 2024). This suggests a more sophisticated model for the pathophysiology of AD, perhaps involving endothelial damage as a precursor to increased permeability for protein and fibrillar tangle deposition, and highlights a potential new avenue for disease modification.

### 1.5 The Eye–Brain Connection

I will now aim to address how the eyes and central nervous system are inherently linked, and explore the evidence for AD pathology being preceded by changes in the eye that can be detected non‐invasively with retinal imaging.

### 1.6 The Eye and the Brain Have the Same Embryological Origins

**FIGURE 5:**
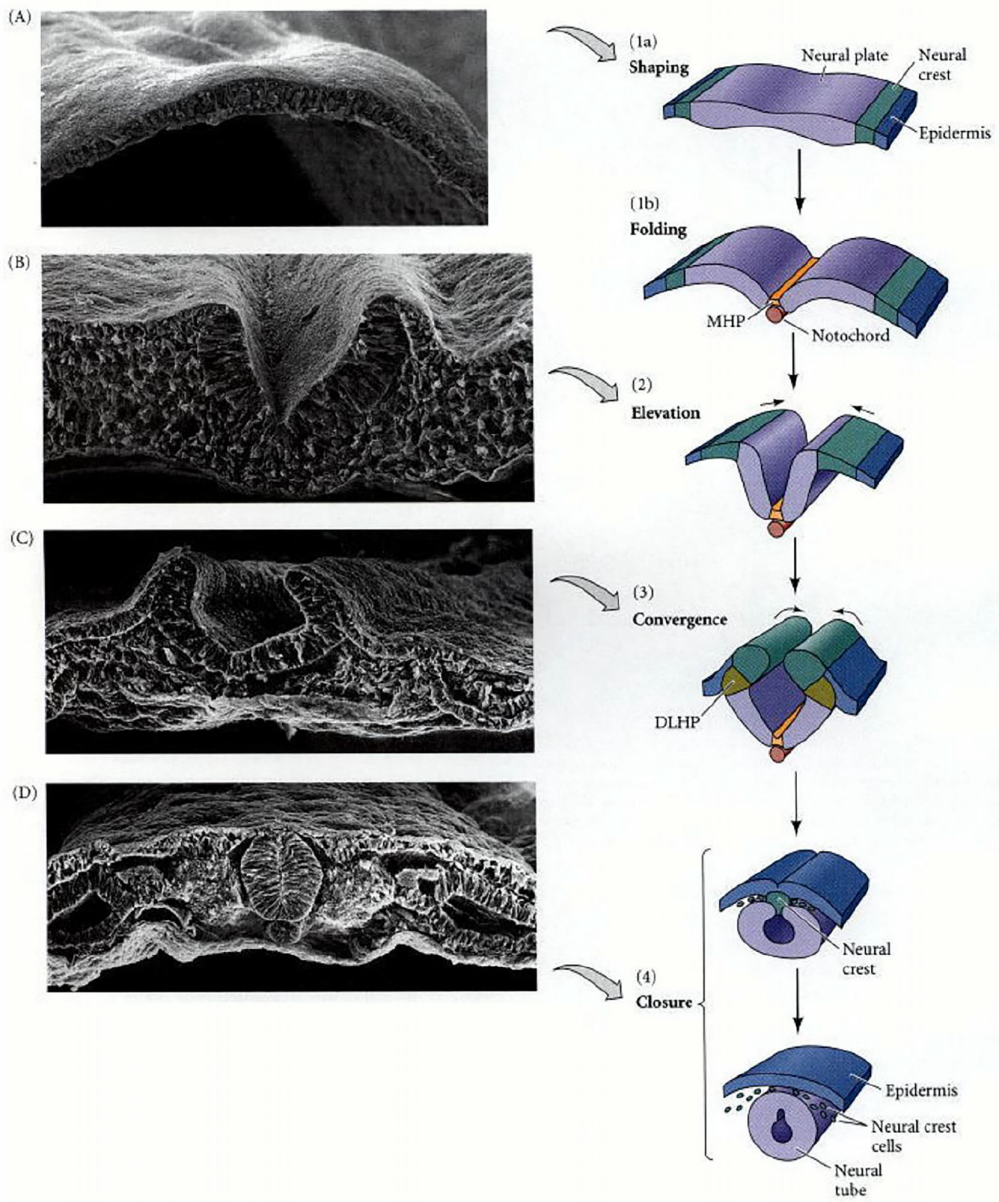
Schematic diagram of the process of neurulation (Gilbert 2000).

Figure 5 gives a brief overview of the process of neurulation, gleaned from studies looking at the Xenopus (Borodinsky 2017) and chick animal models (Schoenwolf 2018).

- (1) is the forming and shaping of the neural plate beginning around week 3 of development, utilizing patterning molecules to ensure the correct orientation. A common molecule between neural tube and retinal vasculature development is Wnt, which works alongside VEGF (vascular endothelial growth factor) to promote and direct vessels to the correct locations. This is evidenced as Wnt knock‐out mice demonstrate a “hypo‐sprouting” phenotype (Selvam et al. 2018).
- (1b) and (2) involve the bending of the neural plate to form a hollow tube using “hinge joints” and the closure of the neural groove to form overlying skin, muscle, and vertebrae.
- (3) highlights the development of neural crest cells, whose delamination from adjacent cells is prompted by a variety of transcription factors. The neural crest cell migration is also central to eye development, and abnormalities in this process can lead to craniofacial and ocular manifestations such as Axenfeld‐Rieger Syndrome and primary congenital glaucoma (Williams and Bohnsack 2015).

The eyes are embryologically similar to the brain, with many derangements in the processing of one inherently impacting the other. This provides an interesting opportunity for neurodegenerative disorders to be imaged in the same way.

### 1.7 Retinal Imaging Shows These Shared Structures

Established retinal imaging techniques include fundoscopy, retinal angiography, and ocular coherence tomography (OCT). Fundoscopy arguably most heavily relies on the shared origins of the brain and eye. It allows direct visualization of the back of the eye, providing information on the macula, optic disc, and cup, which gives information on optic nerve health, and the vascular properties of the eye with hemorrhages, ischemia, and emboli being visible, as described by Hollenhorst in 1961 (Kaufman et al. 2025). OCT was developed in 1991 (Huang et al. 1991) and uses the interruption of coherent waves of light to create an in vivo image of the structural layers of the eye. This is widely used to identify corneal and retinal abnormalities. Both of these techniques can be combined with fluorescein‐angiography to visualize the vascular system of the eye with contrast media. This can identify morphological changes, leaks, or blockages to elucidate pathology.

### 1.8 Retinal Imaging Demonstrates Vascular Differences Between AD Patients and Healthy Controls

Given the accessibility of retinal imaging modalities and the array of information that can be gleaned in relatively non‐invasive ways, studies have begun to use these techniques to investigate links between retinal changes and neurological disease. A meta‐analysis looked at database observational studies that investigated OCT and OCTA retinal images in AD, MCI, and preclinical AD. Of the 26 included articles, results highlighted the significance of the thickness of measured retinal parameters in AD patients, and those with MCI, compared to healthy controls (Table 2) (Ge et al. 2021).

**TABLE 2:** Difference in retinal thickness in those with AD and MCI compared to healthy controls.

	AD patients (*p < *0.001)	MCI patients (*p <* 0.001)
Thickness of peripapillary retinal nerve fiber layer	−0.723	−0.324
Total macular thickness	−0.612	−0.302
Subfoveal Choroid Thickness	−0.888	0.462

Another study looked at the fundoscopic retinal images of patients with AD and healthy controls from the Australian Imaging, Biomarkers and Lifestyle study of aging. ANCOVA analysis of these images revealed larger venular branching asymmetry and an increased arteriolar length‐to‐diameter not only healthy controls found to have an increased amyloid plaque burden, but also in those with established AD. This was in a stepwise manner, with a sensitivity of 76.95% and a sensitivity of 69.25% (Frost et al. 2013).

### 1.9 Non‐Vascular Ocular Changes Also Showed Differences Between AD Patients and Healthy Controls

Researchers in the Indiana Alzheimer Disease Centre used the understanding that many patients with AD have changes in color vision to assess whether contrast sensitivity had measurable differences between cohorts (Risacher et al. 2020). A total of 74 older adults were selected for the study, with 31 of those cognitively healthy individuals and others at various stages of cognitive impairment. In all the participants, poorer visual contrast sensitivity was significantly associated with amyloid and tau deposits as well as temporal lobe volume. This is a relatively small study, and so larger cohorts would be required to make conclusive statements, but these findings suggest visual contrast may be a beneficial screening tool in conjunction to retinal imaging. Another study looked into an association between pupil dilation during cognitive test taking between those with MCI and healthy participants (Kremen et al. 2019). In more than 1000 men aged 56–66 who were enrolled in the Vietnam Era Twin Study of Aging, researchers found that a larger pupil size correlated with a higher genetic risk for AD than those whose pupils were less dilated. It is postulated that this is due to greater cognitive demand in completing the test for those with MCI compared to healthy controls.

### 1.10 Evaluating the Role of AI in Retinal Imaging for Alzheimer's Disease Screening

If our “quadruple aim” remains to improve population health, improve patient experience of care, enhance the caregiver experience, and reduce rising costs (Sikka et al. 2015), then rapidly increasing diagnoses of conditions such as AD in our increasingly elderly population needs novel interventions to help meet those aims. AI has been helpfully described by some as the answer to the growing supply‐and‐demand challenges facing healthcare (Bajwa et al. 2021).

Eye‐AD is an exciting deep‐learning model, designed to integrate OCTA images with what we know of retinal vasculature changes associated with MCI and early‐onset AD to impact diagnostic outcomes (Hao et al. 2024). This model uses a deep learning approach known as multilevel graph representation to assess both the specific features of OCTA images such as vessel and fovea diameter, for example, alongside the relation between these two structures, in an attempt to understand how one factor may influence the other. In this study, OCTA images from multiple centers were used to teach the model what these salient features may be in both MCI and AD cohorts, and how to identify them. Eye‐AD was shown to demonstrate diagnostic accuracy that exceeds three selected models that also use graph neural networks (graph convolutional network, GCN; graph attention network, GAT; and uncertainty aware graph attention network, UG‐GAT), with an accuracy of 0.8885.

Table 3 demonstrates the eight different parameters extracted from the study and the statistical significance of their variance between MCI and AD cohorts. These were extracted to highlight which feature of retinal vasculature is most relevant in AD and MCI detection.

**TABLE 3:** Statistical significance of differences between AD and MCI patients and controls for extracted retinal parameters.

Retinal parameter	Statistical difference between control and AD (*p* value)	Statistical difference between control and MCI (*p* value)
Vascular length density (VLD)	0.074	0.206
Vascular bifurcation number (VB)	0.115	0.935
Vascular fractal dimension (VFD)	0.012	0.443
FAZ roundness (FR)	0.006	0.036

This research not only demonstrates the efficacy of AI modeling in identifying MCI and AD cases in a non‐invasive manner, but also shows that AI mitigates for what we currently don't know will be salient retinal differences between cohorts. This means that while there remains debate on what the exact pathophysiology of AD is, and the role of vascular disease as mapped onto the eye, the machine learning models can help to elucidate novel differences used for diagnosis, and these can in turn retrospectively shed light on the pathology.

This unique advantage is further demonstrated in another study using a framework known as “granular neuron‐level explainer” (LAVA). This is an interpretation model that uses small‐scale inspection of retinal imaging to draw connections to the spectrum of AD without the need for longitudinal studies (Yousefzadeh et al. 2024).

**FIGURE 6:**
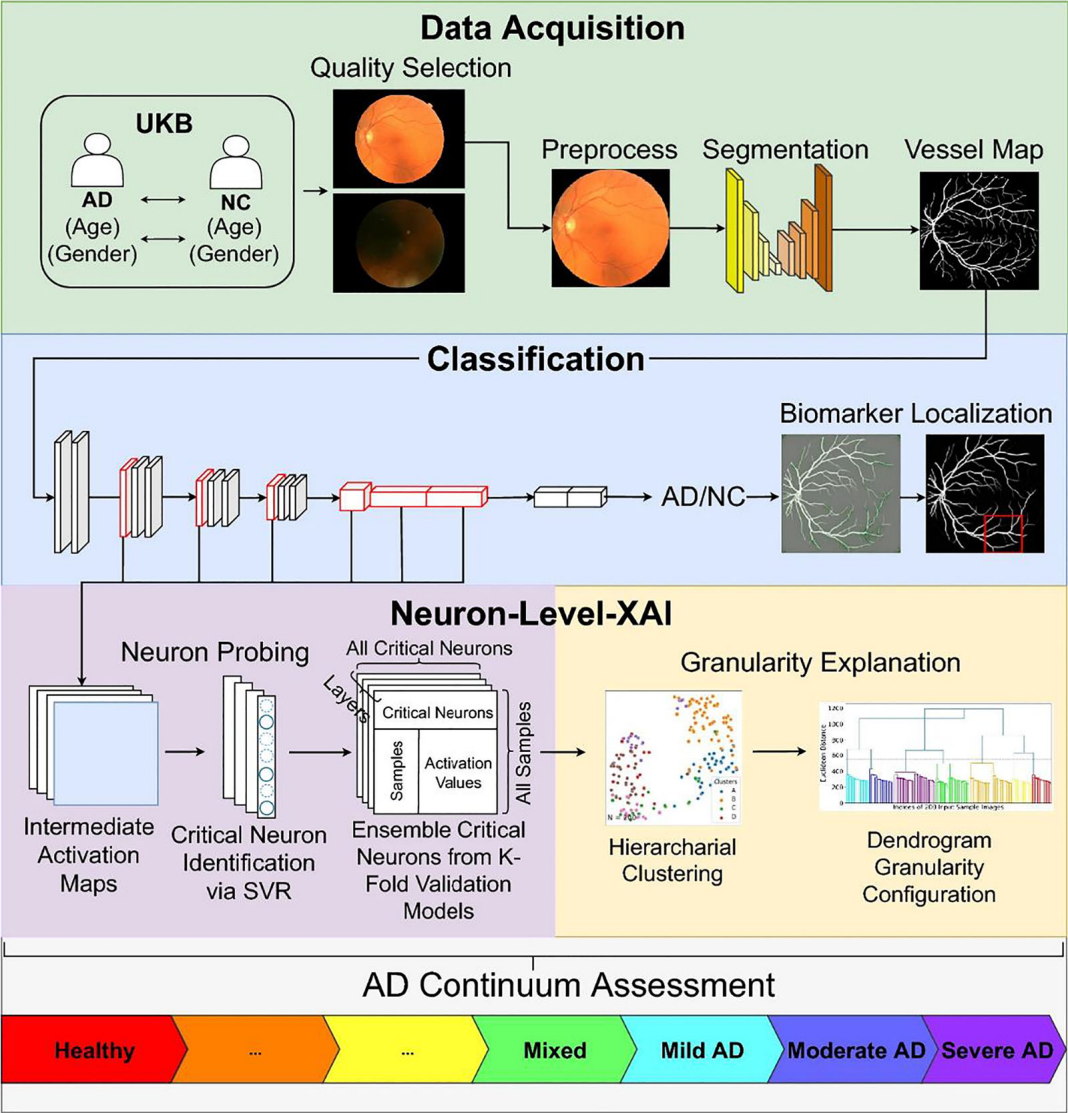
Visual representation of LAVA model to identify AD pathology and salient biomarkers.

This is explained in Figure 6. This model was found to correlate a difference across cognitive level features with morphological features such as vessel density (*p < *0.01), as well as show an ability to determine diagnostic biomarkers and regions of interest between subgroups. This further highlights the role of AI not just in MCI and AD diagnosis, but also in developing our understanding of the pathophysiology of the disease.

This is a growing field as AI's potential in neurological care has shown utility in managing neurological comorbidities in other conditions such as HIV, further demonstrating its versatility in personalized diagnostics (Mugisha et al. 2025).

## 2 Discussion

When proposing any new clinical test, ethical considerations need to be considered particularly on the accessibility of predictive AI models and how earlier diagnoses should be handled. The Wilson and Jungner criteria outline a set of principles required for an effective population‐based screening program.

**FIGURE 7:**
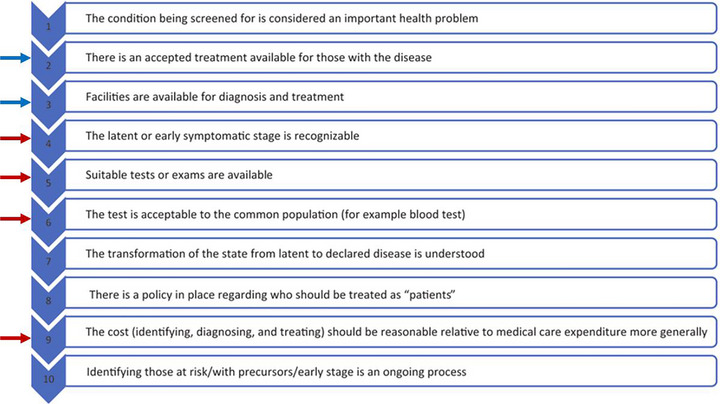
Wilson and Jungner criteria for population‐based screening.

These criteria are outlined in Figure 7, where red arrows indicate the need for the test to be effective, cost‐effective, and non‐invasive, that is, acceptable to the general population; and blue arrows indicate the need for the test to only be implemented if we can already treat the condition.

I hope that so far, I have explained how retinal imaging can become an effective and non‐invasive tool to screen for AD, and that the use of AI modeling can improve its efficacy. However, the criteria requirements for an effective treatment are worth further consideration. In AD, the current mainstay of treatment (acetylcholinesterase inhibitors such as donepezil, rivastigmine, and GABA‐antagonists such as memantine) are not disease modifying. Although there have been exciting emerging therapies such as stem cell treatments being explored for their potential to modify AD progression (Pradhan et al. 2022), it remains to be debated whether an earlier diagnosis at this stage would be more helpful or psychologically harmful to patients and their families. The other important consideration is the feasibility of a large‐scale retinal imaging program to intended marginalized communities. Though non‐invasive, the testing requires some training to correctly interpret images in the face of artifact (Greig et al. 2020), and a financial investment to establish among communities (Song et al. 2021) which may in turn provide a significant barrier, thereby worsening the socioeconomic disparities already seen with the disease. That said, given what has been shown about the potential impact of accessible lifestyle factor modifications to ameliorate the disease, a global commitment to prioritize early diagnostic tools like retinal imaging arguably holds the potential to maximize therapeutic benefits.

## 3 Conclusion

Currently, the diagnosis of AD involves our understanding of the molecular aggregates method of pathology and the functional impact. This involves a clinical assessment, with the use of reputable cognitive screening tests such as the MOCA, imaging techniques to visualize cortical atrophy in primarily the temporal and hippocampal regions, and lumbar puncture p‐tau/tau levels. While these cumulatively provide a reliable basis for diagnosis, often this is a long‐winded process, taking place after symptom onset, where disease modifying agents will have reduced efficacy, and can involve invasive procedures which carry their own risks.

In this essay, I have explored how our understanding of AD has begun to change to involve a heavy role for vascular dysfunction in its pathophysiology. I have also highlighted evidence that these changes can be seen in the eye due to the eye–brain connection.

Retinal imaging offers a non‐invasive and cost‐effective way to detect AD‐related neurovascular changes and, when coupled with AI, holds a transformative role in improving screening, diagnostics, and our understanding of the disease. Though there are important considerations to address such as the utility of an earlier diagnosis for a condition where current treatments are limited and may have deleterious effects in long‐term use, I believe there is enough evidence to suggest that lifestyle modification can be crucial in the progression of the disease, and an earlier diagnosis can allow for meaningful changes to be made.

This creates scope for a non‐invasive test, utilizing a multidisciplinary approach with community opticians, physical therapists, clinical ophthalmologists, neurologists, primary care physicians, and AI modeling working together to optimize screening. I hope that this would increase access to services for known marginalized communities, particularly the elderly, homeless, disabled, and low income. Evidence has highlighted that addressing inequalities in neurological care is critical to ensuring equitable access to such screening tools (Uwishema and Boon 2025), and we know that AD can be a significant manifestation of these inequalities in patient populations if left unaddressed.

## Author Contributions

**Eniola Awodiya**: writing – original draft.

## Ethics Statement

As this is a literature review, no ethical approval was sought from any institution.

## Conflicts of Interest

The author declares no conflicts of interest.

## Peer Review

The peer review history for this article is available at https://publons.com/publon/10.1002/brb3.70890

## Data Availability

Data sharing is not applicable to this article as no datasets were generated or analyzed during the current study.
